# Microbial community structure and source contribution to the early neovaginal microbiota following penile inversion vaginoplasty

**DOI:** 10.3389/fcimb.2026.1816814

**Published:** 2026-05-13

**Authors:** Lili von Bargen, Dhrati Patangia, Nicola von Ostau, Jan Buer, Jochen Heß, Jan Kehrmann

**Affiliations:** 1 Institute of Medical Microbiology, University Hospital Essen, University of Duisburg-Essen, Essen, Germany; 2 Department of Urology, University Hospital Essen, University of Duisburg-Essen, Essen, Germany

**Keywords:** gut, microbiome, microbiota, neovagina, penis, scrotum, urethra

## Abstract

**Introduction:**

The process of colonization of the neovagina in the first year after surgical creation is largely unknown. We characterized the microbiota of the donor tissue sites that formed the neovagina, of the gut and of the neovagina itself to evaluate the source of the neovaginal microbiota.

**Methods:**

Thirty transgender women assigned male at birth were included. Using *16S rRNA* sequencing and aerobic culture, we analyzed the penile, scrotal, urethral and rectal microbiota before surgery, and the neovaginal microbiota in the period of 2 to 13 months after vaginoplasty.

**Results:**

Each of the sites was characterized by a specific microbiota. The neovagina was characterized by the second highest bacterial richness and diversity after the gut. The neovaginal microbiota was dominated by anaerobic bacteria of the phyla Firmicutes and Bacteroidota. The most abundant genera were *Prevotella*, *Peptoniphilus*, *Anaerococcus* and *Porphyromonas*. These genera, together with *Dialister*, *Finegoldia* and *Actinomyces* formed a neovaginal core microbiota, representing genera present in more than 50% of the samples, each with a minimum relative abundance of 1%. More than half of the neovaginal bacteria were not shared with the tissue sites that formed the neovagina. The site with the highest percentage of shared neovaginal Amplicon Sequence Variants (ASVs) was the gut, followed by the urethra. Complementary aerobic culturing showed that on average 38.7% of neovaginal bacterial species were shared with at least one other site.

**Discussion:**

The early neovaginal microbiota is highly diverse, dominated by anaerobic bacteria and is distinct from the microbiota of the tissue sites that form the neovagina. As most neovaginal ASVs are unshared, this indicates that bacteria from other environments may colonize the neovagina in this early period after creation of this new environment. These findings suggest implications for neovaginal health, potentially informing studies on probiotic therapies to support the colonization of the neovagina with beneficial bacteria.

## 1 Introduction

The gut and vaginal microbiota contribute to colonization and infection resistance (Belkaid and Hand, 2014; Chee et al., 2020). The healthy vaginal microbiota in cisgender women is dominated by the genus *Lactobacillus*, while a high diversity of the vaginal microbiota with *Prevotella* and other anaerobic species characterizes bacterial vaginosis (Ravel et al., 2011). Bacterial vaginosis and anaerobic dysbiosis of the vaginal microbiota have been linked to increased inflammation and a higher risk for the acquisition of sexually transmitted infections (Tamarelle et al., 2018; Chee et al., 2020). In addition, bacteria of the genus *Prevotella* of the dysbiotic vaginal microbiota reduce efficacy of tenofovir vaginal gel for the prevention of HIV infection in women who are at high risk for acquiring HIV infection (Klatt et al., 2017). The incidence of sexually transmitted infections is higher in transgender women than in other cohorts (Callander et al., 2019; Van Gerwen et al., 2020; Stutterheim et al., 2021). However, in contrast to the vaginal microbiota of cisgender women, knowledge of the development of the neovaginal microbiota composition of transgender women, its role for neovaginal health and its relevance for the susceptibility to acquire sexually transmitted infections is scarce.

The number of gender-affirming surgeries has increased worldwide (Chaya et al., 2022; Wright et al., 2023; Aksoy et al., 2024). Feminizing genital gender affirming surgery involves surgical reconstruction of the genital organs in transgender individuals to align with their gender identity. Vaginoplasty is an important surgical procedure involving the construction of the neovagina. This can be achieved by penile skin inversion (PIV), or by using intestinal or sigmoid tissues in bowel/sigmoid vaginoplasty. Previous studies investigated the neovaginal microbiota composition after a mean interval of 4.3 to 11.6 years post PIV (Weyers et al., 2009; Kaufmann et al., 2014; Petricevic et al., 2014; Grosse et al., 2017; Birse et al., 2020; Rojas-Vargas et al., 2025) and reported it to exhibit similarities to cis-vaginal bacterial vaginosis (BV), to gut and penile microbes and also to the taxa in oral cavity (Weyers et al., 2009; Birse et al., 2020; Winston McPherson et al., 2024; Rojas-Vargas et al., 2025); few studies report the presence of *Lactobacillus* (Kaufmann et al., 2014; Petricevic et al., 2014), which is the dominating genus of healthy vaginal microbiomes of cisgender women. Studies investigating the neovaginal microbiota from intestinal vaginoplasty report similarities with the microbiota of the colon, reviewed by (Stoehr et al., 2024). Understanding the origin and development of the colonization of the healthy neovagina may facilitate to clarify the role of bacteria in neovaginal health. Data exploring the microbiota of the gut and all tissues involved in the construction of the neovagina are lacking. In the present study, we analyzed the microbiota of the gut, penis, urethra and scrotum on the day of PIV surgery and compared them with the neovaginal microbiota prior to the planned second stage procedure with an average time interval of approximately 4.7 months between the primary construction of the neovagina and the second operation. We used *16S rRNA* gene sequencing along with aerobic culture-based technique to characterize the microbiota of the gut, penis, scrotum, urethra and neovagina from the same individuals. We aim to explore the source of the microbial community composition of the neovagina at an early stage and evaluate, whether it is more strongly shaped by the gut microbiota, by the microbiota of the genital tissue sites (penis, scrotum and urethra) used in neovagina formation, or unshared microbiota not recovered from the analyzed tissues.

## 2 Materials and methods

### 2.1 Study cohort and sample collection

The study was reviewed and approved by the Ethics committee of the Medical Faculty of the University of Duisburg-Essen (22-10784-BO) and was performed in accordance with the latest version of the Declaration of Helsinki. Written informed consent was obtained from all patients before inclusion in the study. The study included a total of 30 transgender women assigned male at birth (AMAB) who underwent PIV surgery in the University Hospital Essen between June 2023 and March 2025. The total surgical procedure was divided into two parts. On the day of the first surgery, separate swabs were taken for microbiota analysis (Zymo Research, Freiburg, Germany) and for aerobic microbial cultures from the urethra, penile skin, scrotum and rectum before PIV. Neovaginal swabs for microbiota analysis and aerobic culture were taken between two and thirteen months after the first surgery, directly before a planned second surgery for vulva construction.

### 2.2 Sample processing

Swabs for microbiota analyses were taken using the DNA/RNA Shield™ Collection Tube with Swab (Zymo Research, Freiburg, Germany) and stored in stabilizing DNA/RNA shield at -80 °C until DNA extraction was performed. DNA was extracted with the ZymoBIOMICS DNA Miniprep-Kit (Zymo Research) with a bead-beating step using the Fast-Prep device (MP Biomedicals, Santa Ana, CA) prior to DNA extraction. Sequencing of the V3/V4 hypervariable region of the *16S rRNA* gene was performed by Novogene (Munich Sequencing Center, Germany) with PE250 Illumina paired end sequencing on the NovaSeq 6000 platform. Preparation control samples and a PCR water sample were used as negative controls.

Amies Agar Gel Transport swabs (Thermo Scientific, Waltham, MA) were used for aerobic culture. Swabs were moistened with sterile physiologic saline before sampling was performed and were processed within 24 hours after sampling. All swabs were plated on the two universal media Columbia blood agar and chocolate agar, MacConkey agar for the selective growth of gram-negative rods and Brilliance Candida agar for selective detection of fungi (all from Thermo Scientific). The scrotal swab was additionally plated on CNA agar (Thermo Scientific), the urethral swab on Thayer-Martin agar, a selective agar for the growth of *Neisseria gonorrhoeae*, and the neovaginal swab on Gardnerella selective agar (all from Thermo Scientific). All agar plates were evaluated for microbial growth after 24 hours and 48 hours. Agar media of urethral swabs were incubated for 72 hours for the improved recovery of *N. gonorrhoeae*. All grown colonies with different morphology were isolated and identified using MALDI-Tof MS. Two different devices (VITEK^®^ MS PRIME, bioMérieux, Marcy L’Etoile, France, or MALDI Biotyper^®^ Sirius, Bruker, Billerica, USA) were used for identification.

### 2.3 Bioinformatic and statistical analysis

Forward and reverse FASTQ files were demultiplexed using the QIIME2 (Quantitative Insights Into Microbial Ecology2) pipeline (Bolyen et al., 2019). Correction and quality filtering of the sequences was performed using the DADA2-package in QIIME2 with filtering of chimeric sequences using the consensus-method. Taxonomy was assigned using the QIIME2 feature classifier plugin for assigning taxonomy with a Naïve Bayes classifier, trained on the SILVA_138_99_ref_seqs of the V3/V4 amplicon sequences. A total of 11 samples (4 scrotal, 4 urethral and 3 penile samples) with less than 10000 quality filtered reads were excluded from subsequent data analyses. All control samples had less than 200 sequences and were thus removed from further downstream analysis. Downstream processing of QIIME2 results was performed in R (v4.5.1) with various packages including phyloseq, ggplot2, microbiome, microbiomeutilities, vegan and complexheatmap. QIIME2 processing resulted in a phyloseq object with a total of 12370 amplicon sequence variants (ASVs) in 138 samples with an average of 41379 reads per sample (minimum reads: 12616, maximum reads: 67276). Mitochondrial and chloroplast reads (Family!= “mitochondria” & Class!= “Chloroplast” & Phylum!= “Cyanobacteria/Chloroplast”) were filtered out, resulting in 11622 ASVs for alpha diversity analysis. Additional filtering and quality control steps were performed for further analyses: Firstly, we performed filtering to retain ASVs with a mean relative abundance more than 10^-5 across samples and present in more than 1% of all samples, resulting in 1282 ASVs. After filtering, there were no NAs at phylum, class, order or family level, 42 NAs at genus level were assigned as Unknown. The filtered phyloseq object with normalized proportions was then used for all further analyses.

ASV level alpha diversity analysis was performed by estimating various diversity indices including the Shannon diversity index, Chao1 richness estimate and Pielou’s evenness index and were visualized with boxplots. The statistical significance for differences in alpha diversity between sites was evaluated as follows: since 11 samples were excluded from subsequent analyses due to low number of reads, samples from all sites were not available for each subject, paired Wilcoxon signed-rank tests were only performed on subjects for whom both sites were available, thus ensuring valid within-subject comparisons. The sample size (N) thus varied by site pair and represents the number of subjects with paired samples included in each comparison (**Supplementary Table 1**). The resulting p-values were corrected for multiple testing using the Benjamini-Hochberg false-discovery rate (FDR). Pairwise comparisons with an adjusted p-value < 0.05 were considered as significant. Differences in the community composition between sites were estimated using Beta diversity analysis through Principal coordinates analysis (PCoA) with various distances (Bray-Curtis, Jaccard, Weighted and Unweighted UniFrac). Statistical significance between groups was assessed by permutational multivariate analysis of variance (PERMANOVA) and pairwise comparisons were performed using pairwise.adonis from vegan package.

Differences in the taxonomic profiles between the sites were determined using pairwise Wilcoxon signed-rank test as detailed above. Core taxa of the neovaginal sample group were determined using the core_members function from the microbiome package in R. ComplexUpset package in R was used to visualize shared and unique ASVs between sites. To assess within subject microbial sharing across anatomical sites, ASVs were identified for each site within each subject. For each subject, ASVs detected in neovagina samples were compared against ASVs detected in gut, urethra, penis and scrotum samples from the same individual. ASVs present in neovagina and at least one other site were classified as shared ASVs, while ASVs detected exclusively in neovagina and not observed in any other site from the same subject were classified as unshared ASVs. Shared ASVs were counted separately for each site, allowing individual ASVs to be shared across multiple sites. As a result, shared ASV counts and percentages are not mutually exclusive and may sum to more than 100% across sites. For each subject, the total number of neovaginal ASVs was calculated, along with the number and proportion of ASVs shared with each donor site and the proportion classified as unshared ASVs. Summary statistics, including minimum, maximum, and mean proportions, were calculated across subjects. Associations between the proportion of shared or unshared neovaginal ASVs and host metadata (BMI and age) were assessed using Spearman’s rank correlation. Differences across categorical variables (sexual practice, active and passive anal intercourse) were evaluated using non parametric tests. For binary variables, Wilcoxon rank-sum test was applied, while Kruskal-Wallis test was used for variables with more than two categories. Resulting p-values were corrected for multiple testing using the Benjamini-Hochberg false discovery rate (FDR).

MaAsLin2 (Mallick et al., 2021) was used to detect differentially abundant taxa with the neovagina as reference group with the following parameters: random effect as Subject ID, normalization=“none” and max_significance=0.05. Top50 coefficients (based on –log10(qval)*sign(coefficient) score) were used for further analysis. All statistical analyses were performed in R and all p-values were adjusted using the FDR method, with adjusted p-values (q-values) used throughout the manuscript. Adjusted p-values <0.05 were considered as significant.

For culture-based analyses, species cultured from non-neovaginal sites were classified as shared with neovagina, if also detected in that subject’s neovagina. For each subject and site, the proportion of species shared with the neovagina was determined. Site-wise means and standard deviations of shared proportions were calculated across subjects. In addition, unique species and genera detected per site type were enumerated across the cohort. Visualization of shared species patterns was performed using bubble plots depicting the frequency with which individual species were shared with the neovagina across subjects and site types.

## 3 Results

### 3.1 Clinical characteristics of the study cohort

We included 30 transgender women AMAB before they underwent gender-affirming surgery. Their mean age at the time of PIV was 33 years (**Table 1**). The mean BMI of the participants was 25.01 at first stage vaginoplasty. According to the WHO BMI classification, 19 participants were classified as normal weight, six as overweight and four as obese (class 1-3), while weight data was lacking for one participant. About 90% of participants reported estrogen hormone therapy. While two participants (6.7%) reported receiving antibiotic treatment within the three months prior to primary PIV surgery, 36.7% of participants reported antibiotic treatment in the three months prior to the second surgery to form the vulva. The mean period between PIV and second surgery was 4.7 months (minimum 2 months and maximum 13 months; sd=2.6).

**Table 1:** Characteristics of participants included in the study.

Metadata	
mean age in years (range)	33.03 (21 - 63)
mean time between first and second surgery (range)	4.67 (2 - 13)
mean BMI at time of first surgery (range)	25.01 (18.52 - 43.83)
Hormone therapy: n (%)	
Estrogen therapy/Hormone therapy	27 (90)
No hormone therapy	2 (6.66)
Data missing	1 (3.33)
Antibiotic use 3 months prior to the first surgery: n (%)	
Yes	2 (6.66)
No	28 (93.33)
Antibiotic use 3 months prior to the second surgery: n (%)	
Yes	11 (36.66)
No	19 (63.33)
Gender of sexual partners in the last 5 years before surgery: n (%)	
Female	3 (10)
Male	10 (33.33)
Male, Female	6 (20)
None	11 (36.66)
Anal intercourse prior to the first surgery: n (%)	
Yes	18 (60)
No	12 (40)
Passive anal intercourse prior to the first surgery: n (%)	
Yes	17 (56.66)
No	13 (43.33)
Penetrative neovaginal intercourse before second surgery: n (%)	
Yes	2 (6.66)
No	28 (93.33)

### 3.2 Neovagina is a diverse microenvironment with a high richness and a distinct microbial composition

**Figure 1:**
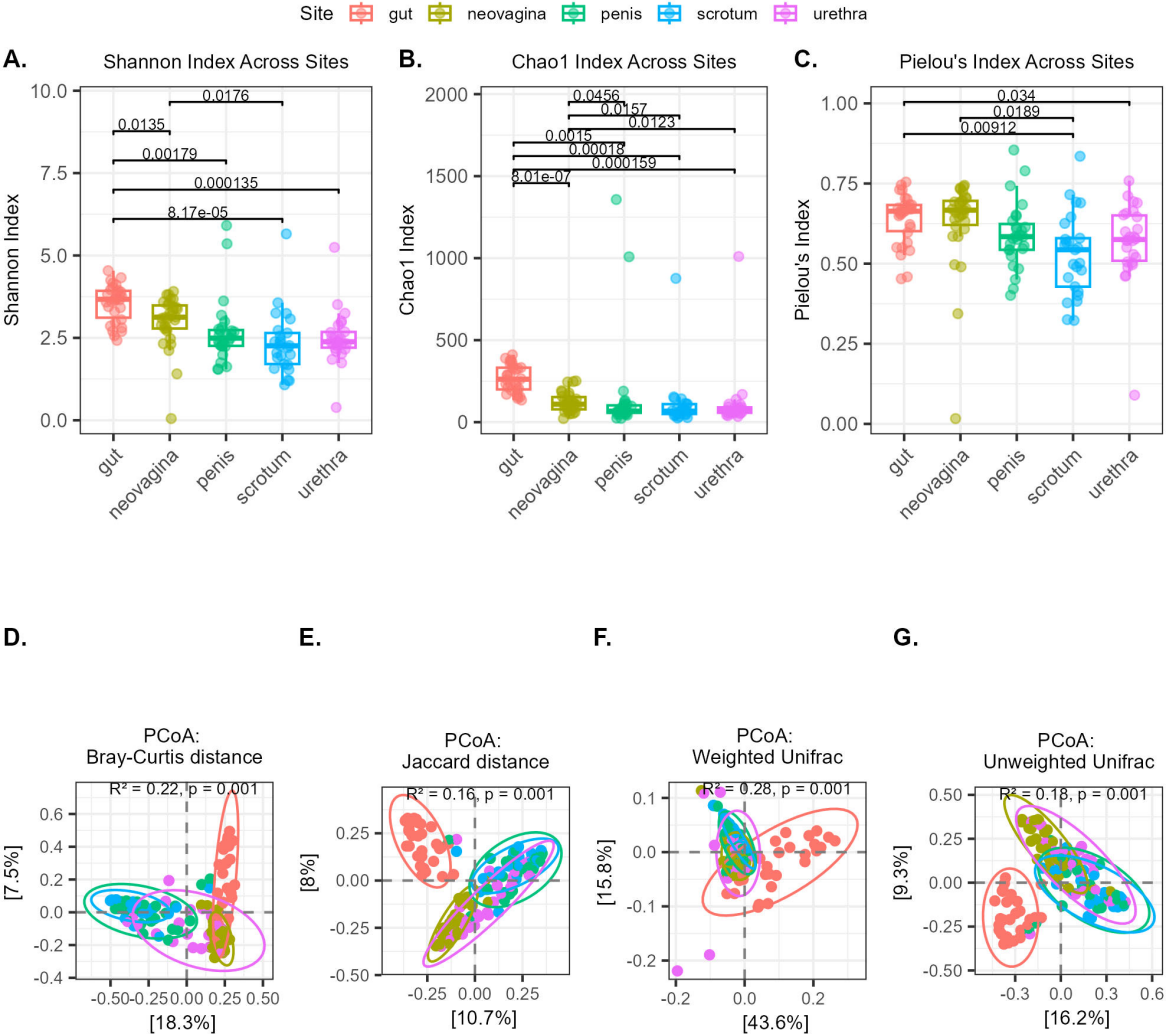
Alpha diversity metrics between sites calculated using **(A)** Shannon diversity index, **(B)** Chao1 richness estimate index and **(C)** Pielou’s evenness index. Beta diversity analysis using PCoA with **(D)** Bray-Curtis distance, **(E)** Jaccard, **(F)** weighted UniFrac and **(G)** unweighted UniFrac distance metrics. PERMANOVA R² and p-values are shown in each panel.

Shannon diversity, Chao1 and Pielou’s evenness index were used to calculate alpha diversity metrics (**Supplementary Table 1**). Shannon diversity of the gut was significantly higher compared to all other sites (p<0.05, **Figure 1A**). Neovaginal Shannon diversity was higher compared to scrotum (p<0.05), while it showed a trend of higher diversity compared to urethra (p=0.07). Based on Chao1 richness estimator, gut followed by neovagina had the highest richness. The richness of the gut and neovagina was significantly higher when compared to all other sites (p<0.05, **Figure 1B**). The gut´s Pielou’s evenness was significantly higher compared to that of urethra and scrotum, and that of neovagina was also higher as compared to scrotum (p<0.05, **Figure 1C**). Furthermore, we evaluated the alpha diversity with respect to antibiotic use in the 3 months prior to the second surgery and found no significant differences (p > 0.05) (**Supplementary Figure 1A**).

Principal coordinates analysis (PCoA) was used to analyze differences in microbial community composition (**Figures 1D–G**). The microbial composition of each site differed distinctly from that of the others (p < 0.05, pairwise PERMANOVA) except between penis and scrotum, as assessed using Bray-Curtis, Jaccard and Unweighted UniFrac. For weighted UniFrac, which incorporates phylogenetic relatedness and relative abundances, all pairwise comparisons were significantly different except between penis and urethra (p=0.059), and penis and scrotum (p=0.538). Overall, beta diversity analysis showed that gut and neovagina microbiota composition were the most distinct from other site types, whereas penis and scrotum communities showed the least differentiation (p>0.05) across all metrics. PERMANOVA using Bray-Curtis and Jaccard distances to determine the impact of antibiotic use in the past 3 months prior to the second surgery showed no significant difference (p>0.05) in microbial composition based on antibiotic use. PCoA was used to visualize the microbial community composition (**Supplementary Figure 1B**).

### 3.3 The neovaginal microbiota is dominated by anaerobic taxa

**Figure 2:**
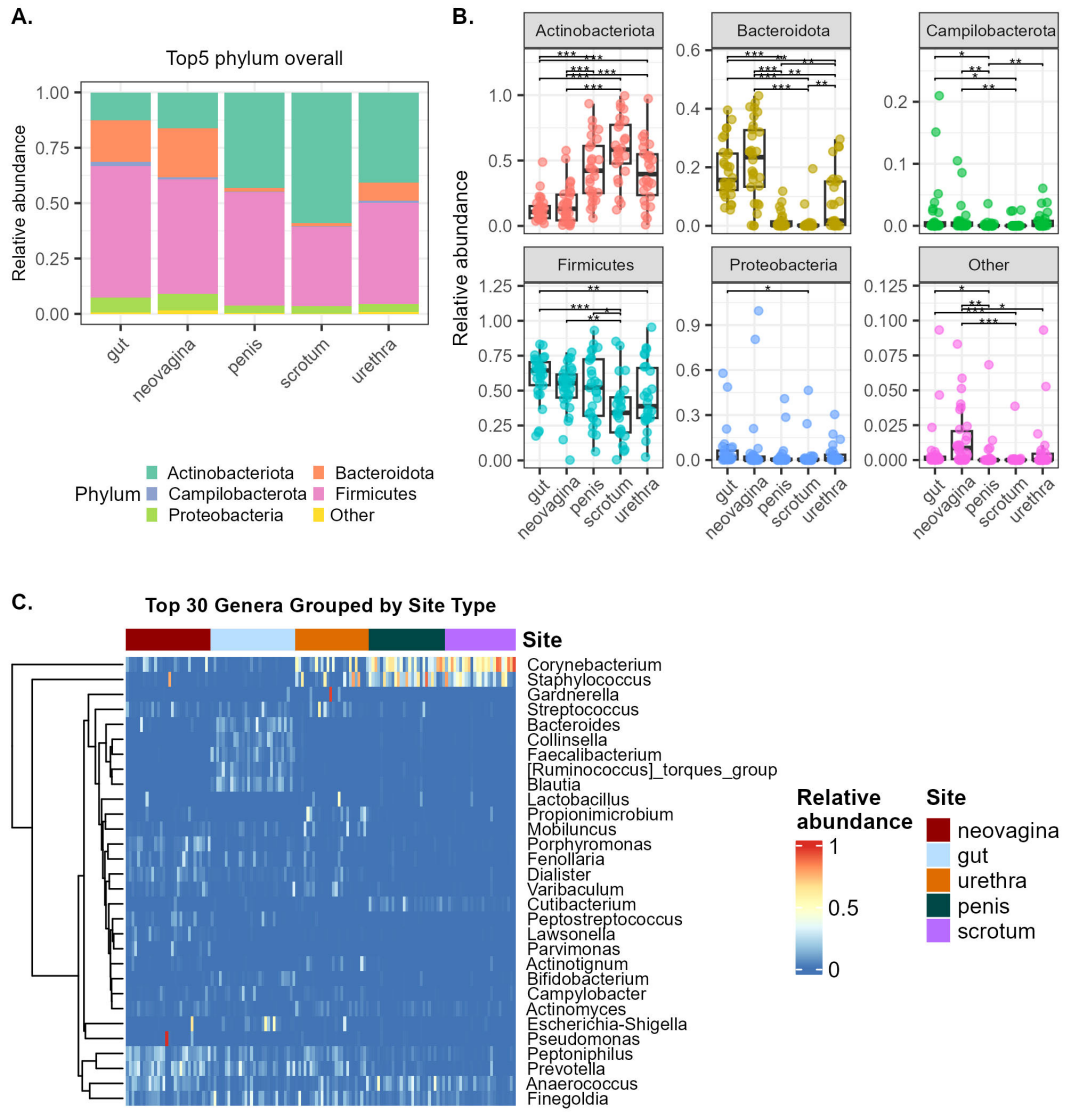
**(A)** Barplots visualizing the relative abundance of the top 5 phyla at all anatomical sites investigated in the study. Other phyla with lower relative abundance were grouped together as “Other” **(B)** Boxplots showing pairwise comparisons between sites for the top 5 phyla. **(C)** Heatmap grouped by site type, exploring the top 30 genera in all samples.

We analyzed the relative abundance of the five most abundant phyla at all sites and grouped together those with a lower relative abundance as “Other” (**Figure 2A**). The phylum Firmicutes was dominant in all sites. The relative Bacteroidota abundance was higher in the microbiota of gut and neovagina compared to all other sites (p<0.05), while that of Actinobacteriota was higher in the microbiota of scrotum, penis and urethra compared to that of the gut and neovagina (p<0.05) (**Figure 2B**).

On genus level, clear differences were observed in the microbiota profiles between the different sites. Of the top30 genera present in all the sites, the gut was dominated by *Bacteroides, Blautia, Collinsella, Escherichia-Shigella, Prevotella, Faecalibacterium* and *Ruminococcus torques group*. Penis, urethra and scrotum were dominated by *Corynebacterium* and *Staphylococcus*. Neovagina samples showed a higher abundance of anaerobic bacteria including *Prevotella, Peptoniphilus, Porphyromonas and Dialister*. The genera *Anaerococcus, Finegoldia* and *Streptococcus* were detected at all sites, but in varying abundances (**Figure 2C**). We performed pairwise multiple testing to determine if any of the top10 taxa were significantly different in the relative abundance in one group over the other (**Supplementary Table 2**). We observed that *Peptoniphilus* was significantly higher and *Staphylococcus* lower in neovagina compared to all other sites (p<0.05, pairwise Wilcoxon test). *Corynebacterium* was significantly lower in neovagina samples compared to penis and scrotum; and *Blautia* and *Bacteroides* were lower compared to the gut. Several other differences between sites were observed as listed in **Supplementary Table 2**. Furthermore, we could only detect *Lactobacillus* in 2 out of the 30 neovagina samples, with relative abundances of 2.3% and 25% in these samples. Antibiotic use in the 3 months prior to the second surgery was not associated with significant differences in the top 10 genera between antibiotic groups (**Supplementary Figure 1C**, Wilcoxon test).

**Figure 3:**
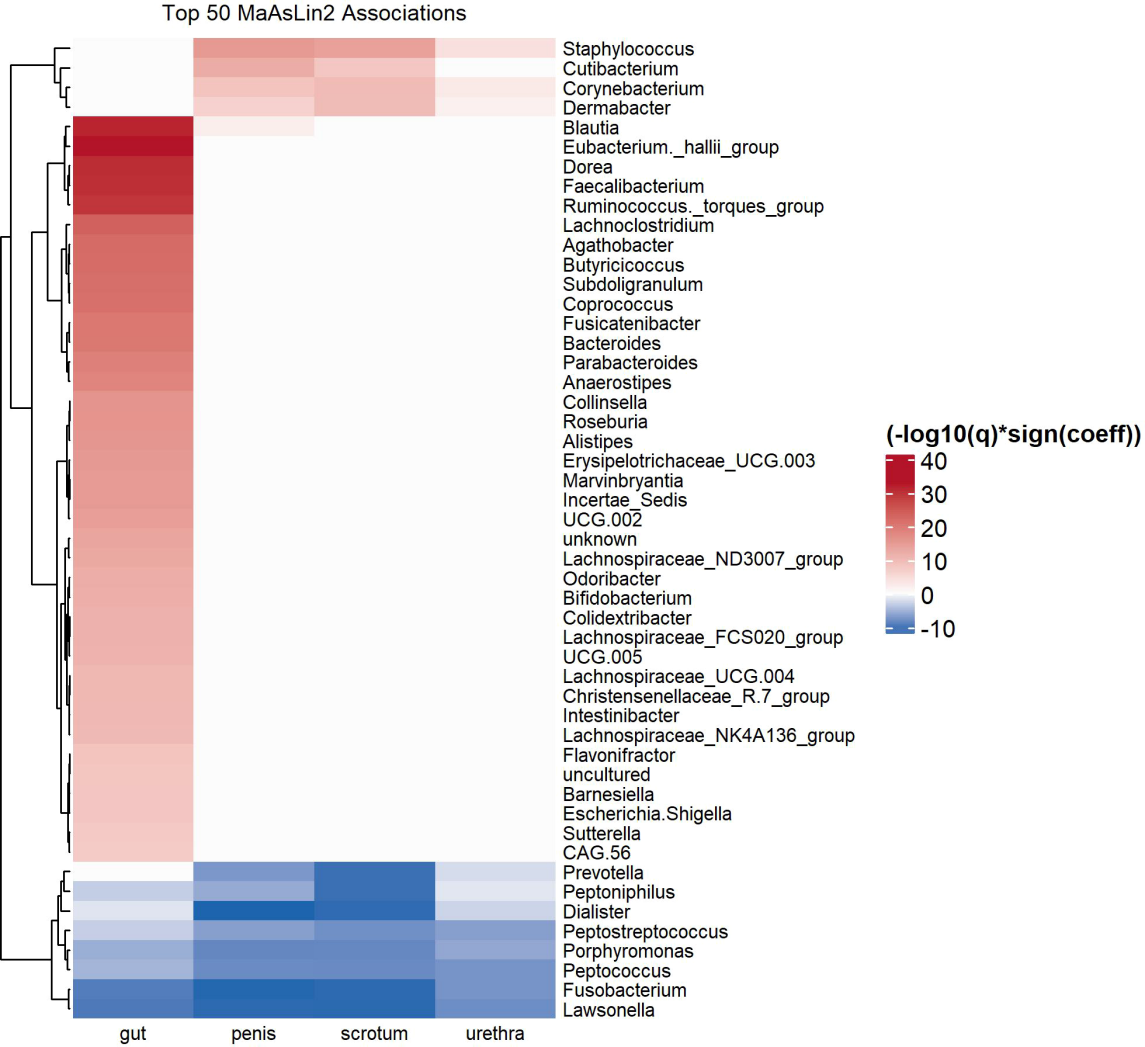
Heatmap showing the top 50 most significant differentially abundant genera over- or underrepresented in the neovaginal microbiota, compared with the other sites, as determined by MaAsLin2 signed –log10(qval)*sign(coefficient) score. MaAsLin2 was run using site as fixed effect and Subject ID as random effect.

Next, we used MaAsLin2 to identify microbial genera over- or underrepresented in the neovaginal microbiota, compared to all other sites, with Subject ID as random effect. Among the top 50 most significant associations, we found *Porphyromonas, Lawsonella Fusobacterium, Peptococcus, Dialister* and *Peptostreptococcus* overrepresented in the microbiota of neovagina compared to the other sites (**Figure 3**). The genera *Blautia*, a genus of *Eubacterium hallii* group*, Faecalibacterium*, a genus of *Ruminococcus torques* group, *Lachnoclostridium* and *Bacteroides* were among the genera that were overrepresented in the gut compared to the neovagina. *Staphylococcus, Corynebacterium and Dermabacter* were overrepresented in penis, urethra and scrotum, while *Cutibacterium* was overrepresented only in microbiota of penis and scrotum.

### 3.4 Core genera of the neovagina, shared and unique ASVs

**Figure 4:**
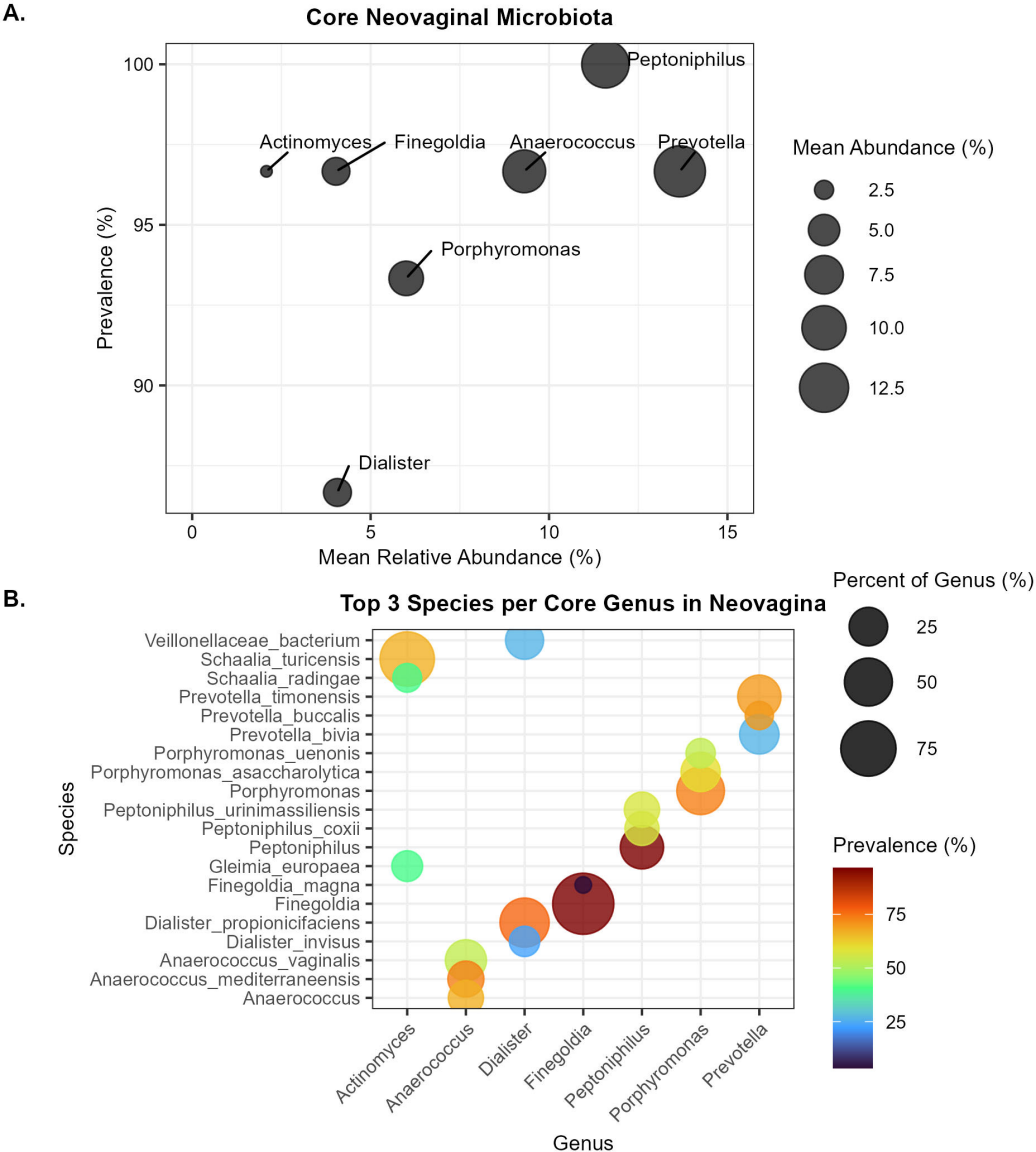
Bubble plots showing **(A)** Mean abundance of core genera (present in at least 50% of the samples with a relative abundance of at least 1%) of the neovaginal microbiota and **(B)** The top 3 species in each of the core genera, showing the prevalence and percentage contribution to their respective genus.

Core members of the neovaginal microbial community were defined as those present in at least 50% of the samples and with a relative abundance of at least 1% as described by Rojas-Vargas and colleagues (Rojas-Vargas et al., 2025). Based on these criteria, we identified 7 genera: *Actinomyces, Anaerococcus, Dialister, Finegoldia, Peptoniphilus, Porphyromonas* and *Prevotella*. Of the core genera, *Prevotella* had the highest mean abundance (13.67%) and was present in 96.7% of neovaginal samples, followed by *Peptoniphilus*, which had a mean relative abundance of 11.6% and was present in all samples (**Figure 4A**). These core genera accounted for a relevant proportion of the neovaginal community, comprising a total of 299 ASVs (23.3%) out of 1282 ASVs; with a median cumulative relative abundance of 58.8% (interquartile range: 39.0–64.7%) across samples. We then looked at the top3 species in each of the core genera and calculated their percent contribution (**Figure 4B**). When species-level identification was not possible due to the limited resolution of *16S rRNA* sequencing, genus names were used. *Prevotella* sp. *timonensis, bivia and buccalis* were the highest contributors in *Prevotella*, all of these species are known colonizers of cis-vaginas (Tett et al., 2021). *Peptoniphilus urinimassiliensis* and *Peptoniphilus coxii* were among the three top members for *Peptoniphilus* and *Porphyromonas asaccharolytica* and *Porphyromonas uenonis* were among those for the genus *Porphyromonas*, however, the species names of the top members of the genera *Peptoniphilus* and *Porphyromonas* could not be assigned by 16S rRNA sequencing. Similarly, *Anaerococcus vaginalis* for the genus *Anaerococcus, Dialister propionicifaciens* for genus *Dialister* and *Schaalia turicensis* for the genus *Actinomyces* were the most abundant species in these genera. The core genera we observed in our study were also part of the top20 genera present in the neovagina samples with *Prevotella, Peptoniphilus, Anaerococcus* and *Porphyromonas* being the most abundant genera (**Supplementary Table 3**).

**Figure 5:**
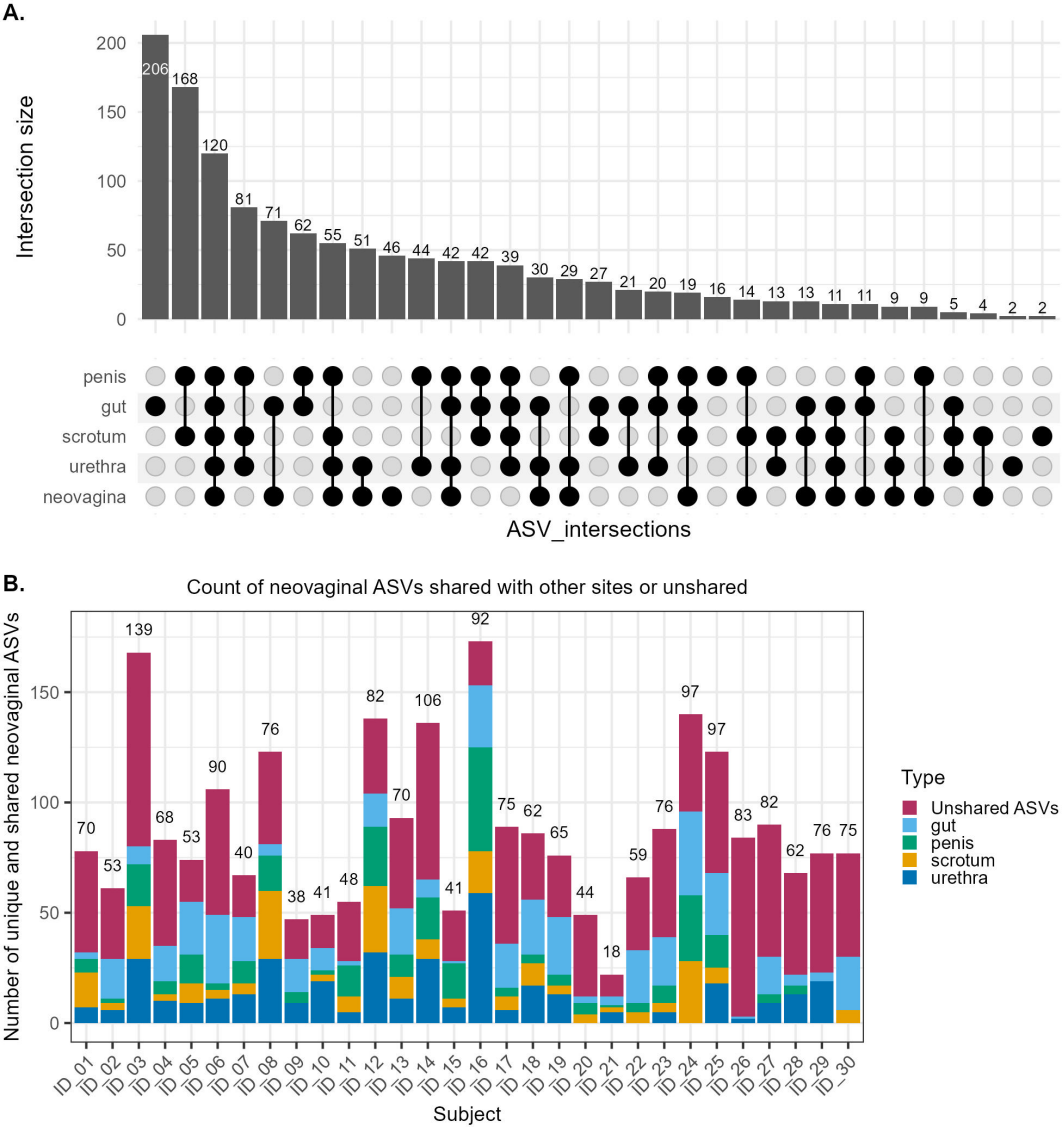
**(A)** UpSet plot showing unique and shared ASVs in all sites of the study and **(B)** Neovaginal ASVs shared with donor sites of each subject (n=30). Colors depict the relative contribution of ASVs from either the donor sites or unshared ASVs to neovaginal ASVs. Stacked bars represent the count of neovaginal ASVs shared with each donor site or unique to neovagina. Numbers above the bars indicate the total number of neovaginal ASVs per subject. ASVs may be shared across multiple body sites; therefore, colored segments are not mutually exclusive and stacked bars may exceed the total number of neovaginal ASVs.

The gut had the highest number of unique ASVs (206), followed by the neovagina (46 ASVs), penis (16 ASVs), urethra and scrotum (2 each) (**Figure 5A**). 120 ASVs were shared between all 5 sites, and many ASVs occurring in limited combinations of groups.

We evaluated how many of the total neovaginal ASVs of each subject were shared with the other sites for that subject, and how many were not detected in any of the other sites for the individual, classified as “unshared ASVs” (**Figure 5B**). The total number of ASVs in the neovagina per subject varied with an average of 70 ASVs (minimum=18, maximum=139 ASVs). Among donor sites, the average proportion of ASVs across subjects shared between neovagina and gut were highest (23.4%), this was followed by urethra (18.9%), penis (14.1%) and scrotum (11.5%). Because individual ASVs could be shared with more than one donor site, percentages across sites were mutually exclusive and did not sum to 100. Donor site contribution to shared ASVs varied widely between subjects. For most subjects, gut was the most common source of shared ASVs, with substantial variations between subjects (ranging from 1.2 to 50%). Urethra showed the highest peak (0 to 64%), this was followed by penis (0 to 51%) and scrotum (0 to 40.8%). A large proportion of ASVs (21.7 to 97.6%) was not detected in any other site and was unique to neovagina and termed as unshared ASVs (**Supplementary Table 4**). No significant associations (p.adjusted < 0.05) were observed between the proportion of shared or unshared neovaginal ASVs and host variables including BMI, age, or reported sexual practices after correction for multiple testing. Although, on average, the gut is the highest contributor of ASVs, between-site microbial sharing is highly individual specific, and may depend on several other factors. These unshared ASVs most frequently belong to the following genera, with *Prevotella* contributing about 9.9% of ASVs*, Porphyromonas* (8.9%), *Peptoniphilus* (7%), *Dialister* (5.8%), *Anaerococcus* (4.6%) *and Corynebacterium* (4.6%).

### 3.5 Microbiota composition determined by aerobic culturing

**Figure 6:**
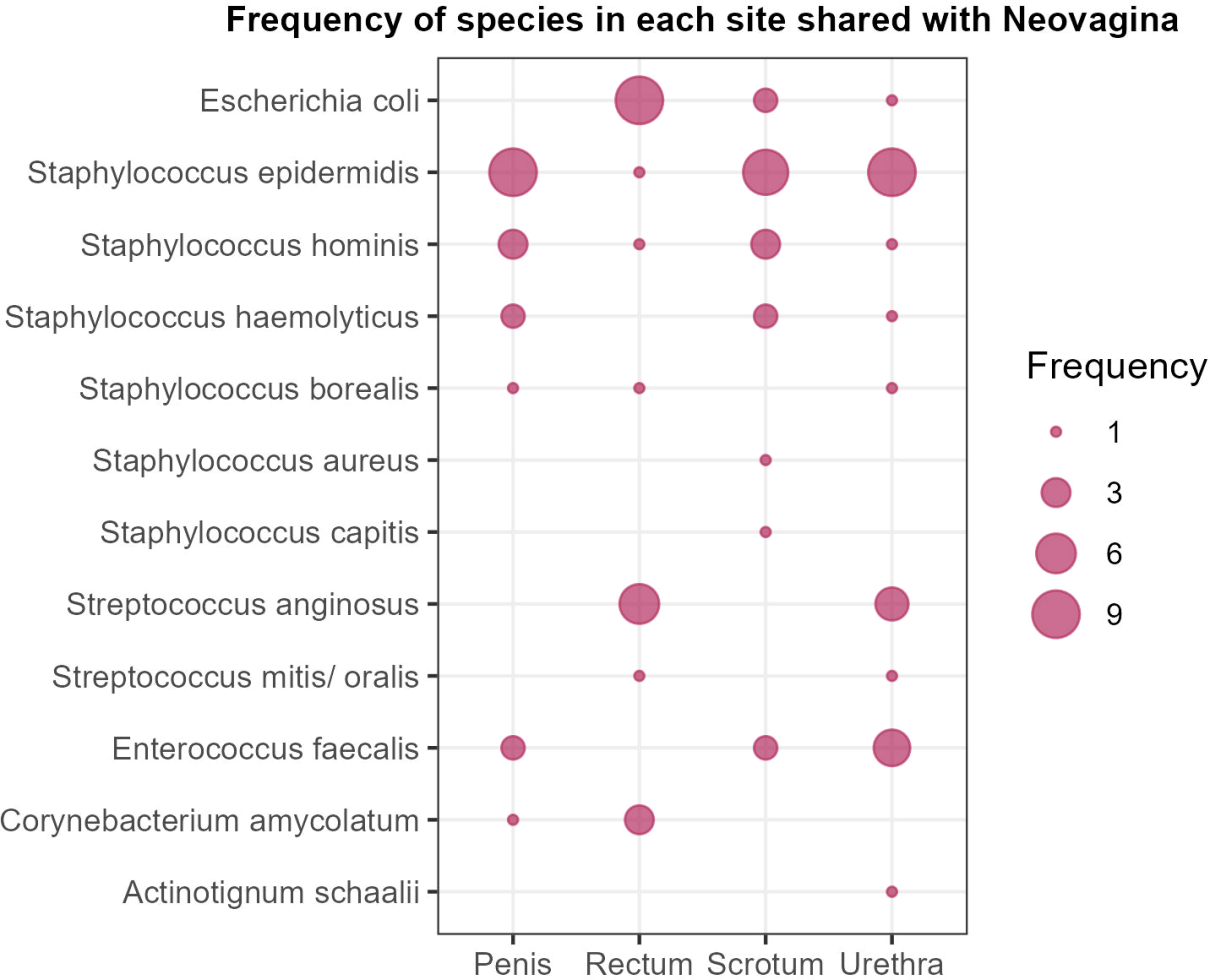
Bubble plot showing microbial species detected by aerobic culture that were shared between the neovagina and other sampled body sites. Each bubble represents a microbial species detected in both, a given site and the subject-matched neovaginal sample, with bubble size indicating the minimum number of subjects showing overlap (range: 1–9 subjects).

Culture-based methods may be advantageous for microbiota characterization compared to *16S rRNA* sequencing for the detection of aerobic pathogens and for identification at the species level, particularly in samples with a high bacterial burden as the gut. We performed aerobic culturing from swabs to study the microbiota from rectum, penis, scrotum, urethra and neovagina. We isolated 119 bacterial isolates of 44 species from the neovagina samples, of which *Streptococcus anginosus, Staphylococcus epidermidis, Enterococcus faecalis*, *Corynebacterium amycolatum* and *Escherichia coli* were the most frequently isolated species from the neovagina. The most commonly isolated species from rectal swabs comprised *E. coli, Streptococcus anginosus, Streptococcus mitis/oralis, Corynebacterium amycolatum, Staphylococcus haemolyticus, Klebsiella pneumoniae, Pseudomonas aeruginosa* and *Haemophilus parainfluenzae*, while the most prevalent species from penis and scrotum swabs included *Staphylococcus epidermidis, Staphylococcus hominis* and *Staphylococcus haemolyticus*. In addition, the most common species grown from urethra swabs were *S. epidermidis, S. hominis* and *Enterococcus faecalis.* We then looked at the species shared between the neovagina and the other localizations in each subject (**Supplementary Table 5**). Of 119 bacterial isolates that were cultured from neovaginal samples, 46 (38.7%) were cultured from at least one other site. Conversely, an average of 19–23% of bacterial species cultured from other sites of a subject were also present in the subject’s neovagina. These species belonged to the genera *Staphylococcus, Streptococcus, Escherichia, Enterococcus, Corynebacterium* and *Actinotignum* (**Figure 6**). As expected, *E. coli* was most frequently shared between subject´s rectal and neovaginal samples, while different *Staphylococcus* spp. were shared between penis, scrotum, urethra and the neovagina. Interestingly, *E. faecalis* overlapped with subjects’ penis, scrotum and urethra samples, but not with the gut. No *Candida* sp. was cultured from the samples included in the study.

## 4 Discussion

The number of feminizing gender affirming surgeries has increased worldwide, with more than a 300% increase from 2006 to 2022 in Germany alone (Aksoy et al., 2024). However, little is known regarding the process of neovaginal colonization and the relative influence of nearby microbiota. Using *16S rRNA* gene sequencing, we investigated the microbiota of the gut, penis, scrotum and urethra in transgender individuals prior to vaginoplasty. We showed that the neovaginal microbiota in the first year after PIV is characterized by a high alpha diversity with the second highest richness behind the gut. It was dominated by anaerobic bacteria and significantly differed from all other tissue sites that formed the neovagina and from the gut. *Porphyromonas, Prevotella, Anaerococcus, Peptoniphilus*, were the most abundant genera, and along with *Dialister* and *Finegoldia* form the major part of a neovaginal core microbiota, defined by genera, present in more than 50% of the samples with a minimum relative abundance of 1%. A vast majority of about 58% of ASVs were not shared with the gut or the tissue site microbiota that formed the neovagina. The site with the highest percentage of shared neovaginal ASVs was the gut with about 23.4% shared ASVs, followed by the urethra with 18.9% shared neovaginal ASVs. Complementing microbiota analyses by using aerobic culture, we found that 38.7% of neovaginal bacterial species were shared with other sites analyzed in this study.

As published studies focused on comparisons of the neovaginal microbiota with the vaginal microbiota of cisgender women and not with the donor tissue site and the gut microbiota of individuals, we cannot directly compare the alpha and beta diversity results with existing studies. However, the gut, together with the oral cavity, is known as the site with the highest alpha diversity in the human body (Human Microbiome Project, 2012). Studies investigating the neovaginal microbiota with a mean of 4.3 and median of 9.5 years after PIV found the neovaginal microbiota exhibits a higher alpha diversity, especially when compared with the vaginal microbiota from cisgender women (Birse et al., 2020; Rojas-Vargas et al., 2025). Skin and urogenital microbiota generally have a lower alpha diversity than the gut (Human Microbiome Project, 2012).

In line with previous studies, the dominant taxa in gut samples in our study were *Bacteroides, Prevotella, Blautia, Finegoldia, Faecalibacterium*, a genus of the *Ruminococcus torques group* (Qin et al., 2010; Arumugam et al., 2011; De Filippis et al., 2020; Neumann et al., 2020; Liu et al., 2021; Shin et al., 2024), while penile microbiota was dominated by *Corynebacterium, Staphylococcus*, *Anaerococcus, Finegoldia and Cutibacterium* (Nelson et al., 2012; Liu et al., 2015; Onywera et al., 2020). The urethral microbiota was characterized by *Corynebacterium, Staphylococcus, Finegoldia* and *Streptococcus* as dominant members, consistent with earlier studies (Dong et al., 2011; Nelson et al., 2012; Hrbacek et al., 2021; Toh et al., 2023). Similar to penile microbiota, scrotal microbiota was characterized by dominance of the genera *Corynebacterium*, *Staphylococcus* and *Anaerococcus*. We observed that the neovaginal microbiota was characterized by a high taxonomic diversity and a unique microbial structure with the core members of the microbiota dominated by anaerobic genera including *Prevotella, Peptoniphilus, Porphyromonas, Anaerococcus, Finegoldia* and *Dialister*. These dominant taxa along with *Lawsonella, Fusobacterium, Peptococcus* and *Peptostreptococcus* were enriched in the neovagina compared with the donor tissue sites and gut. Studies investigating the neovaginal microbiota at later periods after PIV, including a Canadian cohort sampled at a mean of 4.3 years post PIV (Rojas-Vargas et al., 2025), a Brazilian cohort sampled at a median of 9.5 years post PIV (Birse et al., 2020), and an American cohort (Winston McPherson et al., 2024) also reported high abundances of anaerobic taxa including *Porphyromonas, Prevotella, Anaerococcus, Peptoniphilus, Peptostreptococcus, Lawsonella, Dialister*, and *Finegoldia*, that were also overrepresented in the neovaginal microbiota of our study.

In contrast to previous studies, the genera *Campylobacter*, *Fastidiosipila, Anaeroglobus*, *Anaerosphaera* and *Enterococcus* were not dominant genera of the neovaginal microbiota of our cohort. However, our study investigated the neovaginal microbiota within the first 13 months after surgery, while the other studies analyzed the neovaginal microbiota at a mean of 4.3 – 11.6 years post PIV (Weyers et al., 2009; Kaufmann et al., 2014; Petricevic et al., 2014; Grosse et al., 2017; Birse et al., 2020; Winston McPherson et al., 2024; Rojas-Vargas et al., 2025). Of the bacterial species that were cultured from neovaginal samples in our study, many species were also detected in another study, analyzing the neovaginal microbiota by culture, including *Staphylococcus epidermidis*, *Streptococcus anginosus*, *E. faecalis* and *Corynebacterium* spp (Weyers et al., 2009). Additionally, Weyers et al. reported a significantly higher prevalence of *E. faecalis* in microbiota of neovaginal samples from heterosexual transsexual women and those who were sexually active with male partners (Weyers et al., 2009). Due to the small sample size, our study was not powered to formally assess the association between *E. faecalis* and sexual behavior.

Additionally, we did not observe a high abundance of *Lactobacillus* in neovagina samples, consistent with the majority of previous studies where *Lactobacillus* dominance was not a feature of the neovaginal microbiota (Birse et al., 2020; Winston McPherson et al., 2024; Rojas-Vargas et al., 2025). However, Petricevic et al., 2014, reported *Lactobacillus* in 75% of the samples studied (Petricevic et al., 2014) and Kaufmann et al., 2014, recovered lactobacilli in neovaginal samples following a 7 day probiotic intervention (Kaufmann et al., 2014). These discrepancies might be due to several reasons including methodological reasons such as limited sequencing depth, selective PCR approach (PCR-DGGE by (Petricevic et al., 2014)) and probiotic intervention. However, it is noteworthy that most studies investigating the neovaginal microbiota used *16S rRNA* gene sequencing and did not report *Lactobacillus* to be a dominant genus. Another significant factor might be the time interval since vaginoplasty as detailed above and neovaginal physiology and maturation. It is hypothesized that maturation of the neovagina occurs about 12 months post-surgery (Fedele et al., 2006). In the current study, the mean time interval was 4.7 months, which might partially explain the lack of *Lactobacillus* detection in most neovaginal microbiota samples. The physiological environment of the neovagina differs markedly from that of cis-vaginas with the former rich in tissue with keratinized epithelium (Dekker et al., 2007), and therefore might not provide the biochemical conditions to support *Lactobacillus* colonization. Low *Lactobacillus* abundance may be a common feature of the neovaginal microbiota, but its clinical significance and relationship to healthy or dysbiotic neovaginal microbiota remains to be determined.

The dominance of anaerobic bacteria in neovaginal samples, is similar to that reported in pre-pubertal girls, where low estrogen levels and reduced glycogen are associated with diverse microbial communities (Auriemma et al., 2021; Xiaoming et al., 2021). Estrogen-driven pubertal transitions are thought to promote *Lactobacillus* dominance (Hickey et al., 2015); with decline seen again in post-menopausal women (Muhleisen and Herbst-Kralovetz, 2016; Kim et al., 2021; de Oliveira et al., 2022; Chen et al., 2025) with some inconsistencies (Hummelen et al., 2011). These patterns highlight the critical role of age and estrogen associated epithelial and glycogen changes in shaping the vaginal microflora. Owing to the increasing numbers of feminizing genital surgeries and importance of vaginal health it is important that future studies perform neovagina characterizations considering these factors. Several other factors are known to influence the neovaginal microbiota composition including sexual activity (Weyers et al., 2009) and tissues used for neovagina construction (Stoehr et al., 2024). We observed similarities between the neovaginal microbial profile of our study with that reported in uncircumcised penis, which are associated with bacterial vaginosis including the genera *Peptoniphilus, Anaerococcus, Prevotella, Finegoldia* (Price et al., 2010; Liu et al., 2013, 2015; Rojas-Vargas et al., 2025). Though our data provides a comprehensive view of the neovagina microbial community composition with reference to its donor tissue microbiota, our study merits certain limitations. Although the gut demonstrated a high degree of overlap with the ASVs present in neovaginal samples, this association remains correlative, and causality or directional microbial transmission cannot be established based on the current data. Many ASVs present in the neovagina were not detected in samples from the gut or donor tissue sites, suggesting potential contributions from other environments during early colonization. However, limited detection sensitivity, temporal variation, and selective pressures within the neovaginal environment may also contribute to explain the high proportion of unshared ASVs. Microbiota analysis using *16S rRNA* sequencing used in our study has limitations and identification of species is not always possible. However, complementation of neovaginal microbiota analyses using aerobic culture allowed us to investigate the aerobic microbial profile at the species level. Furthermore, functional profiling based on *16S rRNA* sequencing is based on predictive tools and is limited in its resolution, necessitating future studies using shotgun metagenomics sequencing to enable species/strain level characterization and comprehensive functional profiling in this unique microenvironment. Moreover, the neovaginal samples were collected on average 4.7 months after the first surgery, with a range of 2–13 months. These temporal differences may also contribute to the underlying differences in microbial composition. Data about hygiene habits and quantitative hormone levels was not collected, which might also influence the neovaginal microbiota composition.

Our work provides new insights into the early colonization of the neovaginal microbiota showing that a high bacterial richness and a core microbiota, dominated by anaerobic bacteria, already exists within the first year after PIV. However, more than half of the bacterial strains in the neovagina are not part of the tissue site microbiota that form the neovagina with the gut exhibiting the highest number of shared bacteria. Assuming that colonization of newly formed environments is most influenced during the first period after creation, targeted probiotic colonization with *Lactobacillus*, the dominating genus of the healthy vaginal microbiota of cisgender women, might most likely be feasible at early time timepoints after neovaginal creation. A *Lactobacillus* dominated vaginal microbiota is associated with protection against pathogens in cisgender women (Tamarelle et al., 2018; Chee et al., 2020). Nevertheless, it is unclear, whether the lasting establishment of a *Lactobacillus* dominated neovaginal microbiota is feasible and might provide similar beneficial effects compared to those provided to the vaginal microbiota of cisgender women. However, further research with a larger sample size, longitudinal sampling, probiotic interventions, and shotgun metagenomics sequencing will help understanding the role of underlying colonizing bacteria, including *Lactobacillus*, in the neovaginal microenvironment. These future studies will help determine whether the low abundance or absence of *Lactobacillus* in the neovaginal samples represents a typical microbial profile or is due to the altered localized conditions, that may change over time and be benefited by probiotic supplementation. Understanding how the neovaginal colonization is influenced, which species colonize the neovagina and their roles in maintaining neovaginal health, will help to provide better care for transgender women.

## Data Availability

The datasets presented in this study can be found in online repositories. The names of the repository/repositories and accession number(s) can be found below: https://www.ncbi.nlm.nih.gov/, PRJNA1406697.

## Supplementary material

SUPPLEMENTARY FIGURE S1 **(A)** Alpha diversity metrics between antibiotic groups based on antibiotic use prior to second surgery using Shannon diversity index, **(B)** Beta diversity analysis using PCoA with Bray-Curtis distance and Jaccard distance and **(C)** Barplot visualizing the relative abundance of the top 10 Genus in neovagina samples between the antibiotic groups based on antibiotic use in last 3 months prior to second surgery. Other genera with lower relative abundance were grouped together as “Other”.
