# Supplementary figures and images for: Microbial community structure and source contribution to the early neovaginal microbiota following penile inversion vaginoplasty
Source: Front Cell Infect Microbiol. 2026 May 13;16:1816814. doi: 10.3389/fcimb.2026.1816814 (PMC13212302; doi:10.3389/fcimb.2026.1816814)

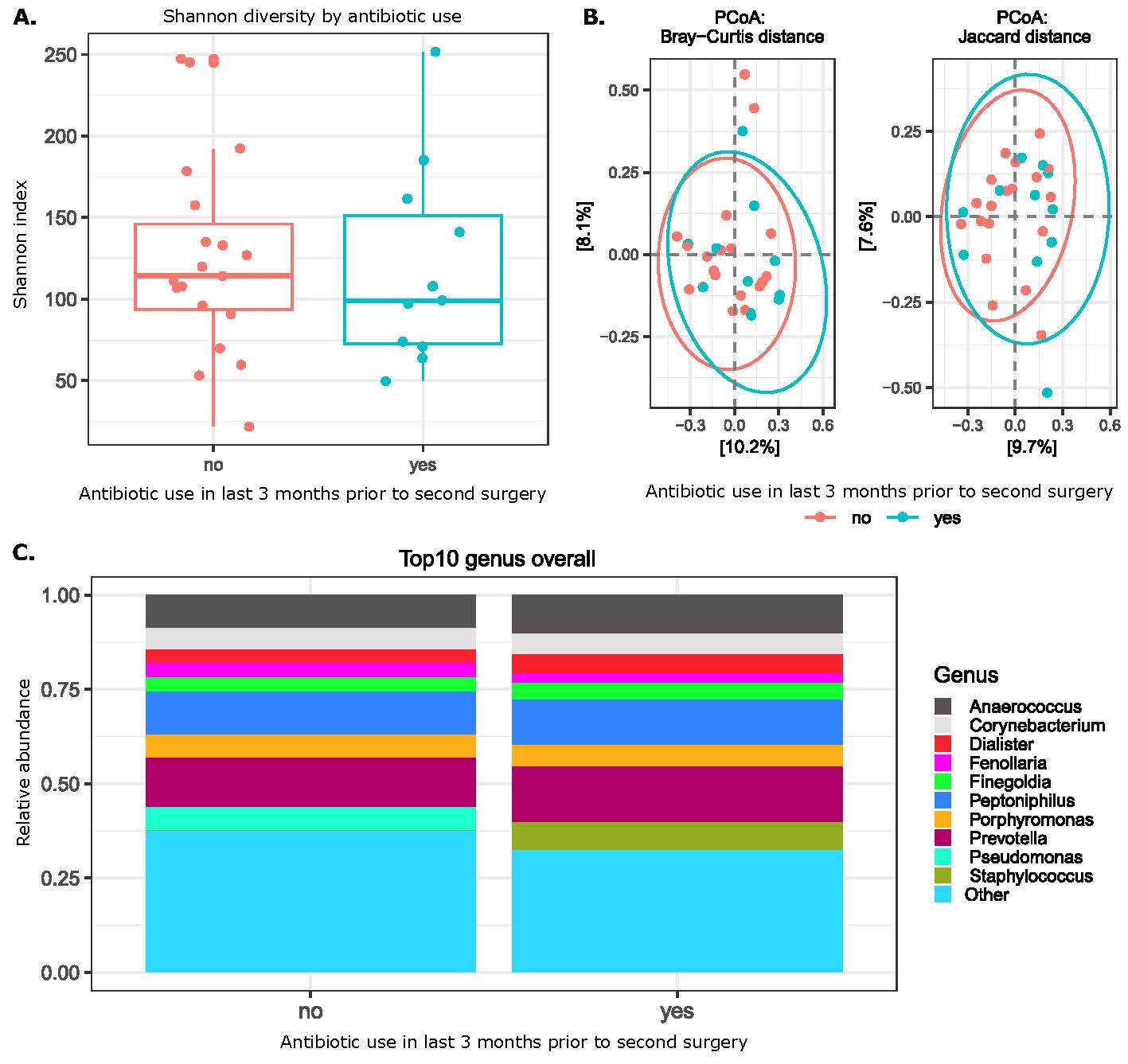

Supplement: SUPPLEMENTARY FIGURE S1 — (A) Alpha diversity metrics between antibiotic groups based on antibiotic use prior to second surgery using Shannon diversity index, (B) Beta diversity analysis using PCoA with Bray-Curtis distance and Jaccard distance and (C) Barplot visualizing the relative abundance of the top 10 Genus in neovagina samples between the antibiotic groups based on antibiotic use in last 3 months prior to second surgery. Other genera with lower relative abundance were grouped together as “Other”. [file Image1.jpeg]
